# A New Concrete Freeze–Thaw Damage Model Based on Hydraulic Pressure Mechanism and Its Application

**DOI:** 10.3390/ma18153708

**Published:** 2025-08-07

**Authors:** Lantian Xu, Yuchi Wang, Yuanzhan Wang, Tianqi Cheng

**Affiliations:** 1 State Key Laboratory of Hydraulic Engineering Simulation and Safety, Tianjin University, Jinnan District, 135 Yaguan Road, Tianjin 300072, China; xlt9903@tju.edu.cn (L.X.); yzwang@tju.edu.cn (Y.W.); 3013205202@tju.edu.cn (T.C.); 2 Tianjin Research Institute for Water Transport Engineering, M.O.T., Binhai New District, 2618 Xingang 2nd Road, Tianjin 300456, China

**Keywords:** concrete, freeze–thaw cycle, freeze–thaw damage model, relative dynamic elastic modulus, air content

## Abstract

Freeze–thaw damage is one of the most important factors affecting the durability of concrete in cold regions, and how to quantitatively characterize the effect of freeze–thaw cycles on the degree of damage of concrete is a widely concerning issue among researchers. Based on the hydraulic pressure theory, a new concrete freeze–thaw damage model was proposed by assuming the defect development mode of concrete during freeze–thaw cycles. The model shows that the total amount of defects due to freeze–thaw damage is related to the initial defects and the defect development capacity within the concrete. Based on the new freeze–thaw damage model, an equation for the loss of relative dynamic elastic modulus of concrete during freeze–thaw cycles was established using the relative dynamic elastic modulus of concrete as the defect indicator. In order to validate the damage model using relative dynamic elastic modulus as the defect index, freeze–thaw cycle tests of four kinds of concrete with different air content were carried out, and the rationality of the model was verified by the relative dynamic elastic modulus of concrete measured under different freeze–thaw cycling periods. On this basis, a freeze–thaw damage model of concrete was established considering the effect of air content in concrete. In addition, the model proposed in this paper was supplemented and validated by experimental data from other researchers. The results show that the prediction model proposed in this study is not only easy to apply and has clear physical meaning but also has high accuracy and general applicability, which provides support for predicting the degree of freeze–thaw damage of concrete structures in cold regions.

## 1. Introduction

The durability of concrete structures during service has attracted a large amount of attention worldwide. Mehta [1], in the Second International Conference on Concrete Durability, analyzed the various data about damage of concrete structures over the past 50 years, summarizing that the main problems of concrete durability are steel corrosion, freeze–thaw damage and physical–chemical effects, which illustrates the importance of studying the effect of concrete freeze–thaw damage. Powers and Helmuth [2,3,4] studied the physical damage mechanism of concrete during freeze–thaw cycles, and their hydraulic pressure theory [2] and osmotic pressure theory [3] are widely used by researchers. The hydraulic pressure theory suggests that freeze–thaw damage is caused by the freezing and volume expansion of pore water within the concrete and the consequent hydraulic pressure. When the water content in the capillary pores exceeds a certain critical value (91.7%), the pore water freezes to generate hydraulic pressure, and the unfrozen pore water migrates from the frozen area to the unfrozen area. The osmotic pressure theory suggests that when a salt solution exists in the concrete pores, freezing of the solution in the large pores will occur first at the same temperature, resulting in an increase in the concentration of the salt solution, which generates a concentration difference with the salt solution in the unfrozen small pores, further generating osmotic pressure, resulting in freeze–thaw damage.

In recent years, many scholars have adopted some microscopic test methods to study the freeze–thaw damage mechanism of concrete more intuitively. Xie et al. [5] investigated and found that many micropores are generated in concrete during freeze–thaw cycles by means of computed tomography (CT) tests and three-dimensional microstructural reconstruction. Yang et al. [6] found that the composition of the hydration products within the concrete remained unchanged with the increase in the number of freeze–thaw cycles, but their morphology changed significantly as tested by scanning electron microscopy (SEM). Zhang et al. [7] obtained the pore distribution characteristics of cement mortar before and after freezing and thawing using nuclear magnetic resonance (NMR) technique. The results show that at the beginning of the freeze–thaw cycle, some of the pores in the mortar produced by freezing and thawing would be filled by hydration products under the continuous influence of cement hydration. With the continuation of freezing and thawing, when hydration is basically completed, the pores in the mortar increase with the enhancement of freezing and thawing effects. Freeze–thaw cycling leads to a change in concrete material from a dense state to a porous state, with micro-cracks appearing in the concrete.

Currently, there are a considerable amount of experimental research studies applying a rapid freeze–thaw cycle test to investigate freeze–thaw damage of concrete. In the rapid freeze–thaw cycle test, the relative dynamic modulus of elasticity is often used to characterize the degree of freeze–thaw damage of concrete [8,9,10,11] as a non-destructive index [12,13] (according to ASTM Standard C666/C666M-15 [14] or the GB/T50082-2009 Standard of China [15]). Wang [16], Liu [17] and other researchers [18,19] conducted experimental investigations on concrete freeze–thaw damage using the rapid freeze–thaw cycle method, of which the results reveal that the relative dynamic elastic modulus of concrete decreases with the increasing number of freeze–thaw cycles. The experiments carried out by the abovenamed researchers provide a large amount of reliable data for researchers.

At present, there are several models to describe the freeze–thaw damage of concrete, which are shown in Table 1. Li [20] proposed an empirical formula to describe the development of relative dynamic elastic modulus of concrete during freeze–thaw cycles. Xiao [21] conducted experiments on the freezing resistance of concrete in a 10% sodium sulfate solution and fitted the experimental data with the empirical formula proposed by Li [20]. It was found that there was a significant deviation between the fitting formula and the experimental data, which indicates that the applicability of Li’s formula [20,21] still needs to be improved. Yu [22] used the relative dynamic elastic modulus of concrete as the damage variable and established an equation based on mechanical fatigue damage theory. The only parameter in his equation is the standard fatigue life of concrete, and the model can predict the fatigue life of concrete relatively accurately, but the accuracy in predicting the extent of damage during freeze–thaw cycles of concrete is insufficient. Wang [23] modified Loland’s concrete damage model [24] and proposed a new model for freeze–thaw damage analysis of concrete. However, the parameter values in the model need to be determined by a large number of physical experiments (e.g., concrete tensile test, resistivity test, microscope scanning test), which are complicated and costly to determine and are limited in practical application. The process of obtaining values of parameters is expensive and complex, which limits the application of this model. Wang [25] considered that the damage of concrete under freeze–thaw cycles could be divided into three stages: rapid growth, slow development and destruction. The freeze–thaw damage model was obtained by fitting the experimental data with the inverse S-type curve. However, the model is an empirical model and lacks theoretical basis, and the accuracy of the model is not clear. Qu [26] concluded that concrete freeze–thaw damage is similar to fatigue damage and used a three-parameter Weibull distribution function to describe the freeze–thaw damage model. The model involves the strength parameter, the determination of which requires a large number of tests. To summarize, most of the models proposed in previous studies lack a theoretical basis and were too complex to be applied or had insufficient accuracy.

The frost resistance of concrete is related to many factors, including the air content of the concrete, the cooling rate, the curing conditions and the type of cement matrix [27]. Concrete in cold regions is generally mixed with an air-entraining agent to improve the freezing resistance of concrete, and the freezing resistance of concrete generally increases with the increase of air content [28]. The principle is that the air-entraining agent introduces a certain amount of tiny bubbles into the concrete, and these bubbles can resist the hydraulic pressure and infiltration pressure generated by the freeze–thaw cycle inside the concrete, so as to keep the development of freeze–thaw damage inside the concrete [29]. Many scholars have studied the durability of air-entrained concrete. Shang et al. [30] found that ordinary strength concrete with an air-entraining agent can also have high freeze–thaw durability. Deng et al. [31] found that the incorporation of air-entraining agents improved the frost resistance of recycled concrete through tests, but the improvement was limited. Hang et al. [32] compared concrete without an air-entraining agent and air-entrained concrete through experiments. There have been predictive models for the degree of freeze–thaw damage of concrete with a single air content in existing studies, but in practice, concrete is mixed with more or less air-entraining agent, and concrete with higher frost resistance requirements is often mixed with more air-entraining agent. Therefore, it is necessary to evaluate the degree of freeze–thaw damage of concrete with different air content to predict the service life of concrete.

In this study, based on the freeze–thaw damage mechanism, the development mode of concrete defects during freeze–thaw cycles is proposed, and a prediction model describing the development of freeze–thaw damage of concrete is established with the loss of relative dynamic elastic modulus of concrete as a variable. The prediction model proposed in this paper was verified by freeze–thaw tests on four kinds of concrete with different air content, using the test data of relative dynamic elastic modulus loss. On this basis, a freeze–thaw damage model for concrete considering the effect of air content was developed. In addition, the accuracy and applicability of the proposed model were discussed based on the experimental data of several other scholars. To summarize, the model established in this study is a theoretical model based on the hydraulic pressure theory, which has the advantages of easy application, clear physical meaning of parameters and good universality, and can provide reliable support for predicting the service life of concrete structures in cold regions.

## 2. A Theoretical Model for Freeze–Thaw Damage of Concrete

### 2.1. Hydraulic Pressure Theory

The hydraulic pressure theory, suggested by Powers [2], is used to explain the mechanism of freeze–thaw damage of concrete. The hydraulic pressure theory considered that freeze–thaw damage of concrete is caused by hydraulic pressure due to volume expansion of internal pore water freezing in concrete. In the process above, the volume of frozen pore water in concrete expands by approximately 9%, causing unfrozen pore water to be forced to migrate outward, under which some new permanent defects appear inside concrete due to hydraulic pressure. Ordinary Portland cement concrete (OPC) has large capillary pores and contains more free water inside. Hydraulic pressure is the driving force for the freeze–thaw damage of OPC in the actual freeze–thaw process [26]. According to the damage mechanism explained by the hydraulic pressure theory, this paper suggests that the main factors affecting freeze–thaw damage of concrete may be the following three aspects:

(1) Hydraulic pressure in concrete: Freeze–thaw damage in concrete is caused by the hydraulic pressure generated by the phase transformation of the liquid phase in the concrete. During freeze–thaw cycles, hydraulic pressure is generated with pore water frozen only under the circumstance that the volume percentage of the liquid phase of concrete is higher than a specific value. Therefore, material properties related to the volume percentage of the liquid phase in concrete (e.g., concrete saturation [33,34,35], water-to-binder ratio [36,37], etc.) have an important effect on freeze–thaw damage of concrete.
(2) Strength of concrete: If concrete strength is not enough to resist hydraulic pressure, new defects will be generated inside concrete. Therefore, material properties related to the strength of concrete (e.g., concrete admixtures [38,39], water-to-binder ratio [36,37], etc.) have an important impact on freeze–thaw damage of concrete.
(3) Initial defects inside concrete: Stress concentration commonly appears at the tips of micro-cracks under hydraulic pressure, so micro-cracks inside concrete can easily extend during freeze–thaw cycles. Therefore, properties of initial defects in concrete (e.g., the number, morphology and distribution of defects inside concrete [2,40,41], etc.) also affect freeze–thaw damage of concrete.

### 2.2. A Freeze–Thaw Damage Model Based on Hydraulic Pressure Theory

This study develops a freeze–thaw damage model based on hydraulic pressure theory for determining the development of defects in concrete.

#### 2.2.1. Theory Parameters

(1) Defects inside concrete (DEF): DEF defined in this model refers to internal material defects, which increase with the number of freeze–thaw cycles.
(2) New defects (NDs): NDs represent the new defects that are generated during one freeze–thaw cycle.

#### 2.2.2. Assumption on Characteristics of Concrete

In the process of an indoor freeze–thaw cycle test, the concrete is immersed in water, and its saturation level will not change significantly. Due to the short duration and low temperature of the freeze–thaw cycle test, the material properties of concrete will not change greatly during this process. Therefore, it is assumed that concrete has the following properties during freeze–thaw cycling:

Assumption 1: Concrete saturation is not changed.

Assumption 2: The material properties of concrete are constant.

The assumptions above ensure that both hydraulic pressure in concrete and the strength of concrete do not change during freeze–thaw cycles, which implies that defects of concrete are the main factor affecting freeze–thaw damage during freeze–thaw cycles.

#### 2.2.3. Mode for Development of Defects Based on Micro-Cracks in Concrete

**Figure 1:**
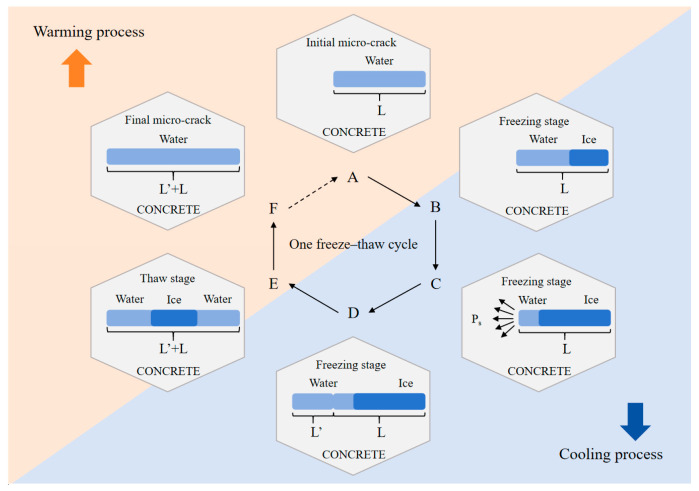
Development mode of defects in concrete.

Figure 1 illustrates the development of a water-saturated micro-cracks defect (initial length is L) in concrete during one freeze–thaw cycle (stages A to F) as an example. A complete freeze–thaw cycle process consists of warming and cooling processes. Firstly, as the external temperature decreases, the water within the concrete micro-cracks is gradually converted to ice from surface to interior, i.e., the A to B stage. At the B–C stage, the phase transition between water and ice causes significant volume expansion of the solid–liquid mixture in the micro-cracks over time, and the unfrozen water in the extruded micro-cracks is forced to migrate outward, which generates hydraulic pressure (Ps) on the solid phase of the concrete. At the C–D stage, due to stress concentration at the tip of the micro-crack under hydraulic pressure, the micro-crack extends along the tip of itself, and the length of the micro-crack increases L’. At the D–E stage, with the increase in the environment temperature, the ice in the micro-crack melts from the surface to the interior, and the volume of the ice–water mixture decreases after the phase transformation. Due to the influence of capillary water absorption, the micro-cracks absorb water from the outside and are filled with water again. At the E–F stage, the environmental temperature continues to increase, and the ice in the micro-crack is completely converted to water. Finally, after the F stage, one freeze–thaw cycle ends. At this point, the micro-cracks in the concrete are still saturated. When the temperature decreases again, the next freeze–thaw cycle continues following the previous process again, and it is worth noting that the initial micro-crack’s length at this point changes to L + L’.

In the process above, it is assumed that the width of micro-cracks does not change, and the volume expansion rate of water into ice is k. When the water inside the micro-cracks completely turns into ice, the length of the micro-cracks will increase by L’ = k × L. According to Powers’ theory [2], the volume of pore water inside concrete expands by about 9% when frozen. However, the pore water cannot completely turn to ice during actual freezing process, and concrete has appropriate strength to resist part of the hydraulic pressure. In reality, the length of the micro-crack will increase L’ = k × L (k < 9%).

The process above implies that if concrete does not have DEF at the initial stage, the concrete will not be damaged during freeze–thaw cycles. In fact, the initial DEF in concrete not only has micro-cracks but also pores and interface transition zones [2,42], all of which are also considered to be consistent with the development pattern of defects in concrete.

#### 2.2.4. Defect Development Equation

Regardless of which form defects may take, it is assumed that the development of all defects in concrete is consistent with the development mode of micro-cracks as outlined above. According to the assumption above and the mode for development of defects, the new defects produced during one freeze–thaw cycle are determined by the total defects accumulated before this freeze–thaw cycle, and there is a linear relationship between ND and DEF, shown by

*ND_n_* = K × *DEF_n_*_−1_ (*n* ≥ 1), (1)

where *ND_n_* represents new defects generated during the nth freeze–thaw cycle, *DEF_n_*_−1_ represents the total accumulated defects within the concrete after *n* − 1 freeze–thaw cycles and K represents the development ability of defects, of which the physical meaning is new defects generated per unit of original total defects. K is mainly related to the strength of concrete. The assumed condition for the concrete material properties mentioned above ensures that K does not change with the number of freeze–thaw cycles.

Supposing there are initial defects (DEF_0_) in concrete, as the number of freeze–thaw cycles increases, the recurrence equations for the development of defects in concrete are as follows:

(2) $$\left\{\begin{array}{l} N=1\;{\mathrm{DEF}}_{0}+ND_{1}=DEF_{1}\\ N=2\;DEF_{1}+ND_{2}=DEF_{2}\\ \;\cdot \cdot \cdot \cdot \cdot \cdot \\ N=i\;DEF_{i-1}+ND_{i}=DEF_{i} \end{array}\right.,$$

where *N* represents the number of freeze–thaw cycles, *DEF_i_*_−1_ represents the total amount of defects in concrete after *i* − 1 freeze–thaw cycles and *ND_i_* represents new defects generated during *i*th of freeze–thaw cycles.

After *n* freeze–thaw cycles, the *DEF_n_* based on Equation (2) is simplified to

(3) $$\;DEF_{n}=\sum_{i=1}^{n}ND_{i}+{\mathrm{DEF}}_{0}\;\left(n\geq 1\right),$$

Based on Equation (1), the relationship between *ND_n_* and *DEF*_0_ can be derived as follows:

(4) $$\left\{\begin{array}{l} ND_{1}=K\cdot {\mathrm{DEF}}_{0};\;ND_{2}=K\cdot DEF_{1}=K\cdot \left({\mathrm{DEF}}_{0}+ND_{1}\right)={\mathrm{DEF}}_{0}\cdot K\left(1+K\right)\\ ND_{3}=K\cdot DEF_{2}=K\cdot \left(DEF_{1}+ND_{2}\right)=ND_{2}\left(1+K\right)={\mathrm{DEF}}_{0}\cdot K{\left(1+K\right)}^{2}\\ \;\cdot \cdot \cdot \cdot \cdot \cdot \\ ND_{n}={\mathrm{DEF}}_{0}\cdot K{\left(1+K\right)}^{n-1} \end{array}\right.,$$

By using Equation (4) to replace *ND*_2_, …, *ND_n_* in Equation (3) with *ND*_1_, the evolution equation for *DEF_n_* characterized by two parameters (*DEF*_0_ and *K*) is obtained:

*DEF_n_* = DEF_0_ (1 + K)*^n^* (*n* ≥ 1), (5)

The evolution Equation (5) indicates that the total amount of defects (*DEF_n_*) in concrete after *n* freeze–thaw cycles are only related to the initial defects and development ability of defects in concrete. Analyzing the expression of this equation reveals that there are two approaches that can be taken to reduce the development of defects in concrete during freeze–thaw cycles: reducing initial defects (DEF_0_) inside concrete and cutting down the development ability of 0 defects (K). DEF_0_ is related to the microstructure of concrete, and K is mainly related to the strength of concrete. Therefore, the frost resistance of concrete can be enhanced by improving the microscopic pore structure of concrete and increasing the strength of concrete. The two methods are consistent with the fact that adding an air-entraining agent into concrete can significantly increase the frost resistance of concrete by improving the microstructure of concrete, and high-strength concrete has high frost resistance [43,44], which proves the rationality of the evolution equation to some extent.

### 2.3. Explanation of the Freeze–Thaw Damage Model

The freeze–thaw damage model is proposed based on the hydraulic pressure theory, which only considers the damage mechanism of the hydraulic pressure hypothesis and does not consider the influence of other freeze–thaw damage mechanisms. Therefore, the model cannot be used to describe the freeze–thaw damage process controlled by other freeze–thaw damage mechanisms, which is a limitation of the model.

The freeze–thaw damage model does not define the indicator to measure the defects of concrete. Researchers can select indicators according to their own investigations, as long as the indicators can reflect the development of defects in concrete during freeze–thaw cycles. Due to the complexity of the initial microstructure within concrete, it is difficult to select microscopic indicators of concrete defects. And there are few studies on which microscopic features in concrete can be considered defects and how to quantify the microscopic defects. From the macroscopic view, defects inside concrete can be characterized by some macroscopic mechanical indicators (e.g., concrete dynamic elastic modulus, compressive strength, etc.). Although macroscopic indicators are less accurate than microscopic indicators in describing the defects inside concrete, they have the advantage of being easy to conduct research and simple for engineering applications.

Ideally, the tests selected for freeze–thaw damage indicators should be inexpensive, rapid, easy to perform and consistent and repeatable [42]. The determination of the relative dynamic elastic modulus is a non-destructive test, which makes it a widely used parameter to measure the frost resistance of concrete. In the following sections, this paper selects the loss of relative dynamic elastic modulus as the indicator to measure the defects of concrete. And an equation for the loss of relative dynamic elastic modulus is established based on the freeze–thaw damage model.

## 3. An Equation for the Loss of Relative Dynamic Elastic Modulus Based on Freeze–Thaw Damage Model

The concrete relative dynamic elastic modulus (*E_r_*) refers to the ratio of current dynamic elastic modulus to initial dynamic elastic modulus.

(6) $$E_{r}=\frac{E_{dn}}{E_{d0}},$$

where *E_d_*_0_ represents initial dynamic elastic modulus of concrete and *E_dn_* represents the dynamic elastic modulus after n freeze–thaw cycles.

The loss of relative dynamic elastic modulus refers to the difference between relative dynamic elastic modulus before and after freeze–thaw cycles, of which the definition is

(7) $$P_{n}=\left|E_{r_{n}}-E_{r_{0}}\right|,$$

where *P_n_* represents the loss of relative dynamic elastic modulus after *n* freeze–thaw cycles, *E_rn_* represents the relative dynamic elastic modulus after *n* freeze–thaw cycles and *E_r_*_0_ represents the initial relative dynamic elastic modulus undamaged by freeze–thaw cycles, of which the value is 1.

At present, many researchers regard the loss of relative dynamic elastic modulus of concrete as the indicator of freeze–thaw damage inside concrete. This paper supposes that there is a positive correlation between the defects generated during n freeze–thaw cycles (*DEF_n_* − *DEF*_0_) and the loss of relative dynamic elastic modulus ($\left|E_{n_{r}}-E_{r_{0}}\right|$). The positive correlation is

(8) $$P_{n}=\left|E_{r_{n}}-E_{r_{0}}\right|=C\cdot ({DEF}_{n}-{DEF}_{0}),$$

where C represents the loss of relative dynamic elastic modulus (%) caused by each additional unit of new defects in the concrete, DEF_0_ is the total amount of initial defects and *DEF_n_* is the total amount of defects after *n* freeze–thaw cycles.

**Figure 2:**
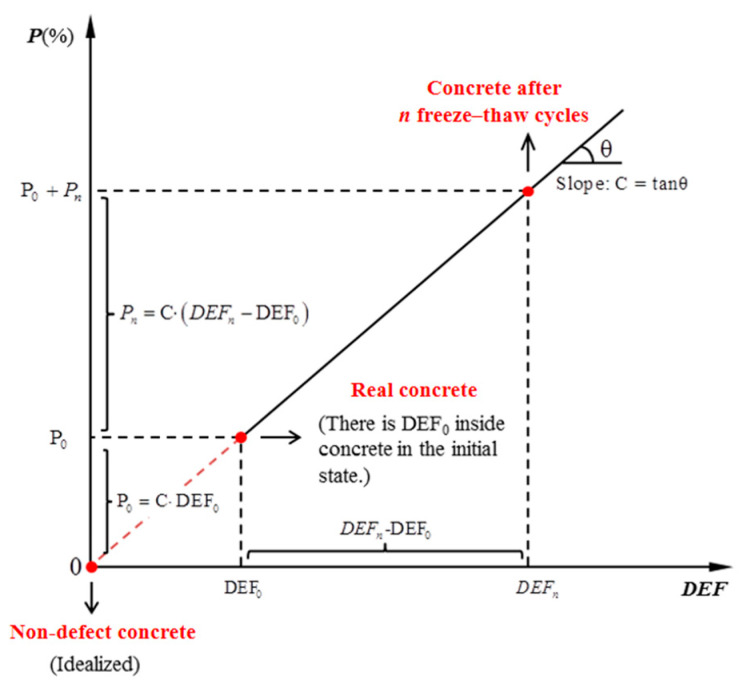
The relationship between defects of concrete and the loss of relative dynamic elastic modulus.

The diagram of Equation (8) is shown in Figure 2. The state in which the number of defects equals zero (DEF = 0) represents the idealized non-defect state of concrete. In fact, the concrete with initial defects (DEF_0_) is the real situation after preparation. When the initial defect is DEF_0_, the corresponding relative dynamic elastic modulus loss rate of concrete is P_0_. P_0_ represents the initial loss of relative dynamic elastic modulus that is generated during the concrete preparation from a non-defect state (DEF = 0) to the initial state (DEF = DEF_0_).

By substituting *DEF_n_* in Equation (5) into the evolution Equation (8), an available equation for the loss of relative dynamic elastic modulus (Pn) is obtained:

*P_n_* = C (*DEF_n_* − *DEF*_0_) = C∙DEF_0_ ((1 + K)^n^ − 1) = P_0_ ((1 + K)^n^ − 1) (9)

Equation (9) is called the relative dynamic elastic modulus loss equation in the following sections. The relative dynamic elastic modulus loss equation characterizes the development of concrete relative dynamic elastic modulus loss with the freeze–thaw cycling process and indicates that the loss relative dynamic elastic modulus of concrete during freeze–thaw cycles increases exponentially with the increase in the number of freeze–thaw cycles.

## 4. Experimental Investigation for the Loss of Relative Dynamic Elastic Modulus of Concrete

In order to investigate the reasonableness of Equation (9), the relative dynamic elastic modulus loss values of concrete with different amounts of air-entraining agent during freeze–thaw cycles were tested experimentally in this paper.

### 4.1. Materials and Preparation

Concrete specimens with a 0.45 water–cement ratio were prepared to investigate the frost resistance of concrete. The mix proportion of concrete is shown in Table 2. Ordinary Portland cement was P.O 42.5 from Tangshan, China, and the density was 3100 kg/m^3^. The fine aggregate was river sand with a fineness modulus of 2.61 and an apparent density of 2660 kg/m^3^. Natural gravel with particle size ranging from 5 to 20 mm was used as coarse aggregates, and the apparent density was 2690 kg/m^3^.

Three types of concrete with SJ-2 (saponin type) air-entraining agent were also prepared in this test, with air contents of 2.7%, 4.4% and 6.6%, respectively. The air content of freshly mixed concrete was measured by a CA-3-type air content meter (manufactured by Beijing Luda Road Industry Testing Equipment Co., Ltd., Beijing, China). The air content of OPC without air-entraining agent was determined to be 0.8%.

**Figure 3:**
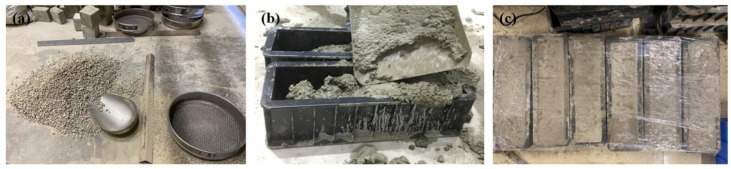
(**a**) Sieving coarse aggregate; (**b**) molds being filled with the mixture; (**c**) initial setting of concrete.

In order to minimize experimental errors, three parallel samples were prepared for each of the four groups of concrete. Figure 3 shows the process of preparing the concrete specimens. Besides the concrete specimens for measuring air content, the rest of the concrete specimens were cast in plastic molds with dimensions of 100 mm × 100 mm × 400 mm. Subsequently, the concrete samples were moved to the curing environment with 20 ± 3 °C and 90% relative humidity. After curing for 24 h, the molds were removed and then concrete samples were placed in saturated Ca(OH)_2_ solution, in which the environmental temperature was maintained at 20 ± 3 °C. After 28 days, all the specimens were taken out and subjected to a freeze–thaw test and a relative dynamic elastic modulus test.

### 4.2. Test Methods

**Figure 4:**
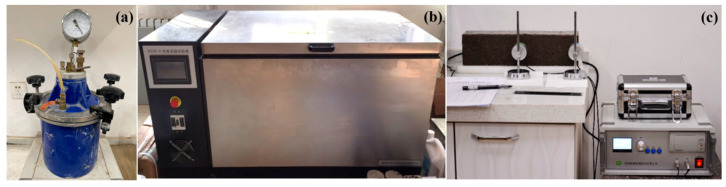
Experimental instruments: (**a**) CA-3 air content meter; (**b**) HDK-9 concrete freeze–thaw testing machine; (**c**) dynamic elastic modulus tester.

An air content determination test was conducted according to GB/T50080-2016 [45] to determine the relevant provisions of air content in the experiment. Figure 4a shows the CA-3 direct-reading air content tester for measuring the air content of concrete.

After 28 days of concrete curing, all the specimens were placed in an HDK-9 concrete freeze–thaw testing machine (manufactured by Donghua Test Instrument Co., Ltd., Suzhou, China), and the concrete was subjected to standard rapid freeze–thaw cycle tests under the condition of distilled water, as illustrated in Figure 4b. The freeze–thaw resistance of concrete was tested according to the GB/T50082-2009 Standard of China [15]. This standard method is similar to ASTM Standard C666/C666M-15 [14]. During the freeze–thaw cycle, the center temperature of the sample was controlled between −18 ± 2 °C and 5 ± 2 °C. The statistical results show that one freeze–thaw cycle lasted 2 h and 58 mins, in which the time for freezing was 1 h and 48 min and the time for thawing was 1 h and 10 min. After reaching a certain number of freeze–thaw cycles, the specimens were taken out of the freeze–thaw testing machine to determine their dynamic elastic modulus. After the completion of the test, the specimens were put back into the freeze–thaw testing machine to continue the freeze–thaw cycle test.

The dynamic elastic modulus of concrete was tested with a digital elasticity modulus tester (manufactured by Jinan Langrui Testing Technology Co., Ltd., Jinan, China), as shown in Figure 4c. The freeze–thaw cycle test was ended when the relative dynamic elastic modulus of concrete dropped to 60% of the initial relative dynamic elastic modulus or the number of freeze–thaw cycles reached 300.

### 4.3. Test Results and Analysis

**Figure 5:**
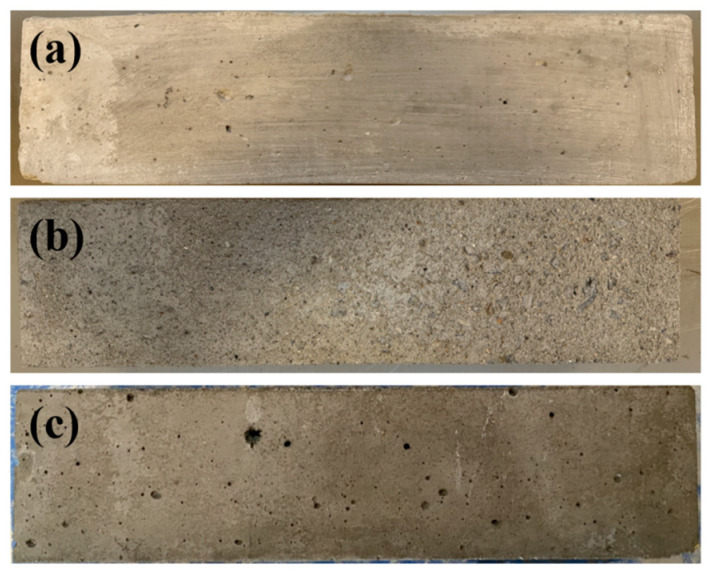
Appearance of concrete specimen before and after freeze–thaw: (**a**) No freeze–thawed concrete; (**b**) ordinary concrete after 125 freeze–thaw cycles; (**c**) 4.5% air content concrete after 125 freeze–thaw times.

Figure 5 demonstrates the appearance of concrete specimens before and after freeze–thawing. In particular, Figure 5a shows the appearance of concrete that has no freezing and thawing, Figure 5b presents the appearance of concrete after 125 freezing and thawing cycles of OPC concrete, and Figure 5c illustrates the appearance of concrete with air content of 4.5% after 125 cycles of freezing and thawing. It is clear from the comparison that after 125 cycles of freezing and thawing, the surface of OPC concrete was no longer flat and smooth but became rough, and the exposed coarse aggregate can be seen in some places. On the other hand, the surface of the concrete with 4.5% air content after the same number of freezing and thawing was still smooth and intact but with some small holes.

**Figure 6:**
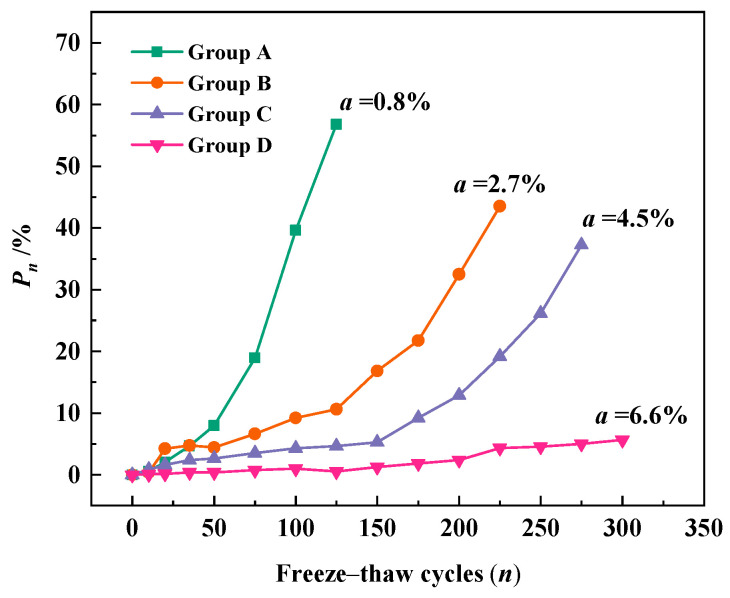
Relative dynamic elastic modulus loss during freeze–thaw cycles.

Figure 6 demonstrates the relationship between the number of freeze–thaw cycles and the relative dynamic elastic modulus loss of concrete. The letter *a* in the figure represents the air content of concrete. As shown in Figure 6, the loss of relative dynamic elastic modulus (*P_n_*) increases with the number of freeze–thaw cycles. And with the increase of the number of freeze–thaw cycles, the growth rate of *P_n_* gradually speeds up, i.e., the loss of relative dynamic elastic modulus accelerates. The reason for this phenomenon is that the damage caused by each freeze–thaw cycle exists in the form of cumulative damage [46], and the higher the number of freeze–thaw cycles, the more serious the damage caused to the concrete. In addition, *P_n_* decreases with increasing air content for the same number of freeze–thaw cycles. At 100 freeze–thaw cycles, the relative dynamic elastic modulus loss of normal concrete reached 40% and the relative dynamic elastic modulus loss of concrete with 2.7% air content was 9%, while the relative dynamic elastic modulus loss of concrete with 6.6% air content was only 1%. This indicates that the addition of an air-entraining agent to concrete can substantially improve the frost resistance of concrete, and the frost resistance of concrete increases with the increase of air content in concrete. It is due to the tiny bubbles introduced by the admixture, which can block the original continuous capillary channels in the concrete, and these bubbles will not be filled by water through the capillary action, which alleviates the internal stress concentration of the concrete when it is frozen [47].

**Figure 7:**
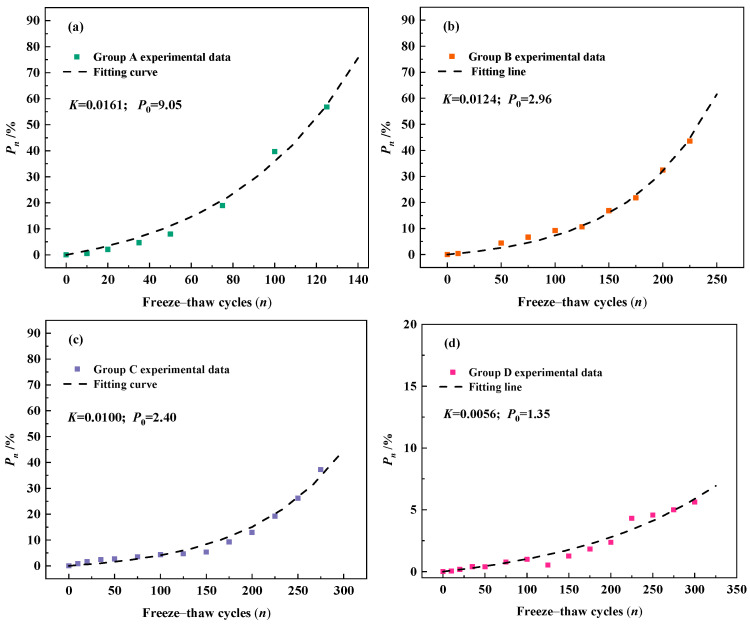
Development pattern of P_n_ in concrete specimens with different air content during freeze–thaw process: (**a**) 0.8% air content; (**b**) 2.7% air content; (**c**) 4.5% air content; (**d**) 6.6% air content.

The relative dynamic elastic modulus loss Equation (9) based on the freeze–thaw damage model was used to fit the development of *P_n_* of concrete with different air content. Regression fitting of the experimental data by the least squares method was performed to obtain the values of K and P_0_ for each group of concrete. Figure 7 demonstrates the fitting results for the four groups of concrete. It can be clearly observed that the fitting results are better for both OPC concrete and concrete with an air-entraining agent, which indicates that the relative dynamic elastic modulus loss Equation (9) proposed in this paper can well describe the development of *P_n_* during freeze–thaw cycles. The model is applicable to both OPC concrete and air-entrained concrete for characterizing the development of relative dynamic elastic modulus loss during the freeze–thaw process.

Table 3 demonstrates the results of fitting specific parameters and the fitting correlation for the four groups of concrete. As shown in Table 3, the development ability of defects (K) and the initial loss of relative dynamic elastic modulus (P_0_) of air-entrained concrete are smaller than those of OPC concrete, and the larger the air content of the concrete is, the smaller P_0_ and K become. As previously analyzed, the addition of an air-entraining agent in concrete improves the frost resistance of concrete, and the introduced air holes not only block the capillary channels but also reduce the stress concentration during freezing. Therefore, the incorporation of air-entraining agents reduces both the initial defects and the ability of defects to develop within the concrete.

### 4.4. Relative Dynamic Elastic Modulus Loss Prediction Model of Concrete Considering the Effect of Air Content

**Figure 8:**
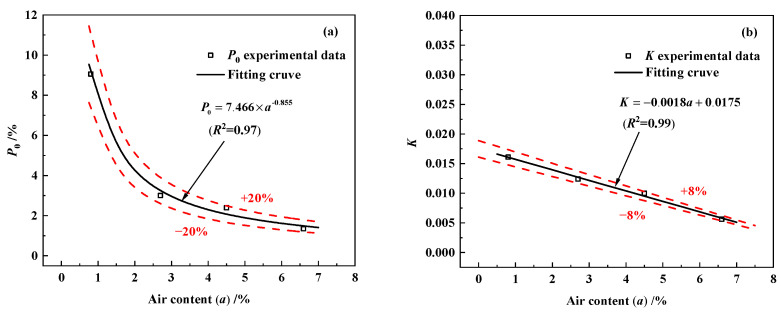
Variation of *P*_0_ and *K* with air content a: (**a**) *P*_0_; (**b**) *K*.

From the analysis in Table 3, it can be seen that the values of *K* and *P*_0_ decrease gradually with the increase in air content. The relationship between the parameters *K*, *P*_0_ and air content *a* in concrete was analyzed, and the scatter correspondences of the data for the two parameters with *a* are shown in Figure 8.

As summarized in Figure 8, *P*_0_ and *K* decrease significantly with the increase in air content *a*. An exponential function was used to fit the initial relative dynamic elastic modulus loss *P*_0_, and a linear function was used to fit the defect development capacity *K* of the concrete. The variation rule of the two parameters with air content was brought into Equation (9), and the relationship between the rate of loss of initial relative dynamic elastic modulus and the number of freeze–thaw cycles considering the change in air content of concrete is obtained, as presented in Equation (10):

(10) $$\left\{\begin{array}{l} P_{n}(a)=P_{0}({\left(1+K\right)}^{n}-1)\\ K=-0.00166a+0.018,\\ P_{0}=10\cdot e^{\left(-0.367a\right)} \end{array}\right.$$

where *n* is the number of freeze–thaw cycles, *a* is the air content of concrete (%), *P_n_*(*a*) denotes the relative dynamic elastic modulus loss rate (%) of concrete with an air content of *a* after *n* freeze–thaw cycles, *K* represents the ability of the concrete to develop defects and *P*_0_ is the loss of relative dynamic elastic modulus due to initial defects generated in the preparation of the concrete.

**Figure 9:**
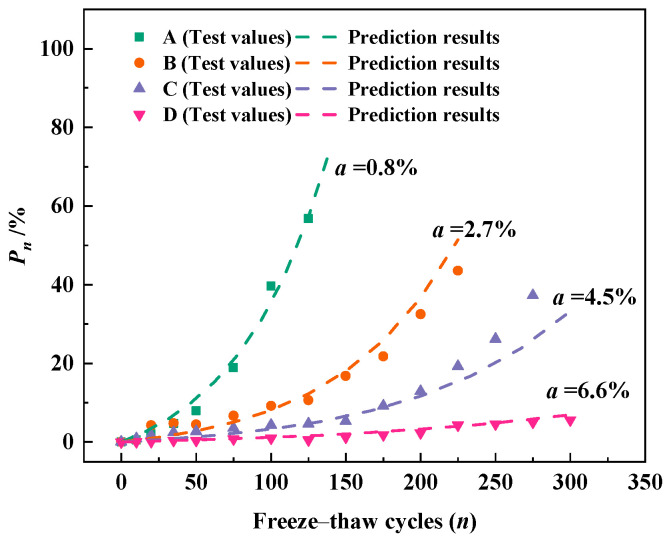
Comparison of Pn between the predictive values and the measured results.

**Figure 10:**
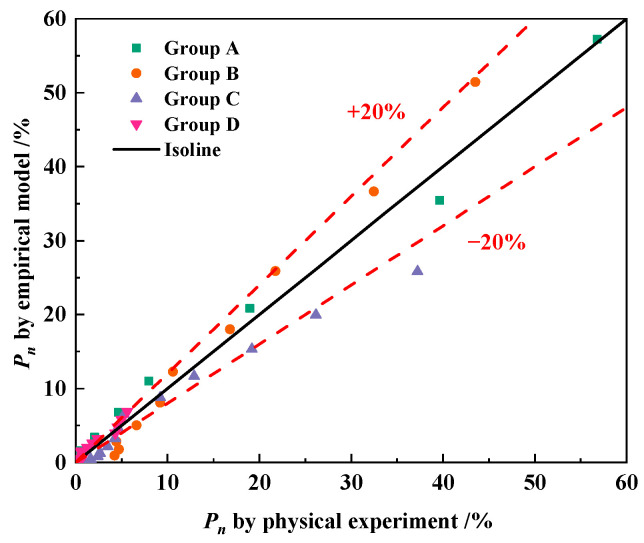
Relative error analysis of the model.

The predictive results of relative dynamic elastic modulus loss *P_n_* under different freeze–thaw conditions can be obtained by substituting corresponding freeze–thaw cycles *n* and air content *a* into Equation (10). As demonstrated in Figure 9, the correctness and credibility of Equation (10) are verified by comparing the predictive values with the measured results. As shown in Figure 10, the relative error between most of the measured values and model predictions can be controlled within ±20%.

## 5. Additional Validation of the Relative Dynamic Elastic Modulus Loss Equation

In order to further illustrate the rationality and applicability of Equation (9), experimental data from freeze–thaw experiments conducted by some scholars were selected to validate the equation. Wang [46], Xiao [48], Zeng [49] and many researchers [18,21,50,51,52,53] conducted physical experiments on the relationship between the relative dynamic elastic modulus of different types of concrete under different freeze–thaw environments and the number of freeze–thaw cycles. Using Equation (7), the relative dynamic elastic modulus test data measured by the researchers above were converted into the loss of relative dynamic elastic modulus (*P_n_*) to calculate the damage of concrete during the freeze–thaw cycle. The converted data for the loss of relative dynamic elastic modulus are shown as follows in Table 4.

**Figure 11:**
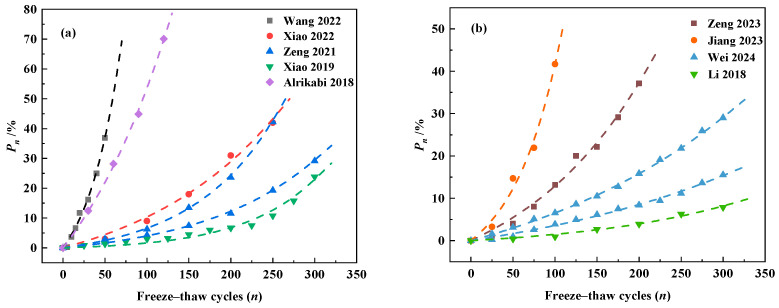
Measured and predicted values of the loss of relative dynamic elastic modulus for researchers’ data [18,21,46,48,49,50,51,52,53]: (**a**) Water freeze–thaw conditions; (**b**) salt freeze–thaw conditions.

**Figure 12:**
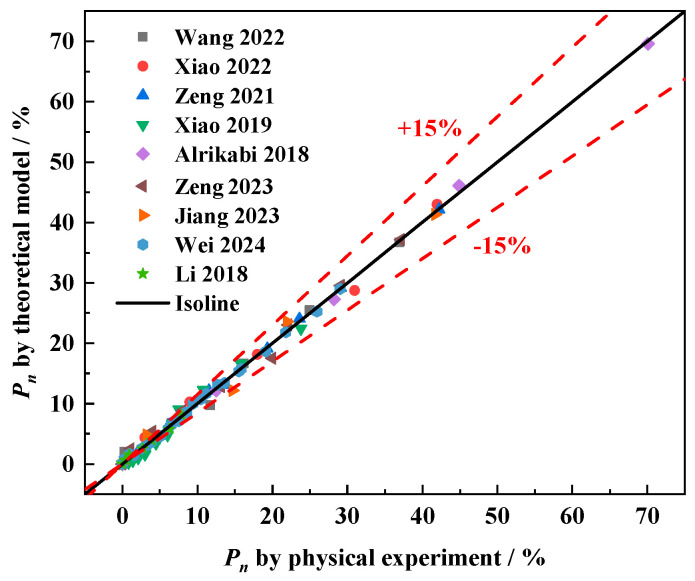
Relative error analysis of the theoretical equation [18,21,46,48,49,50,51,52,53].

The test data of the relative dynamic elastic modulus of the researcher were fitted using Equation (9), and the results are shown in Figure 11. As can be seen in Figure 11, Equation (9) describes well the development of relative dynamic elastic modulus loss of different types of concrete during the freeze–thaw cycles in both water and salt freeze–thaw environments, which indicates that Equation (9) has excellent applicability to different kinds of concretes under different freeze–thaw conditions. And the good applicability of Equation (9) also verifies the rationality of the freeze–thaw damage model proposed by this study. Figure 12 illustrates the prediction error of the relative dynamic elastic modulus loss equation. As illustrated in Figure 12, the relative error between most of the measured values and model predictions can be controlled within ±15%. It proves that the freeze–thaw damage model established in this paper using the relative dynamic elastic modulus loss as an index has good reliability and can be used to predict the damage of concrete during freeze–thaw, which can play a role in predicting the durability of concrete in cold regions.

Table 5 shows the value of the initial relative dynamic elastic modulus defects (P_0_) and the defect development capacity (K) within the concrete in relative dynamic elastic modulus loss Equation (9) based on the abovenamed researchers’ experiments. As seen in Table 5, the result reveals the variation of K is small, roughly in the range of 0.001–0.050, while P_0_ varies widely, roughly in the range of 0.1–100(%). This result provides a reliable reference for parameter analysis for other researchers.

Combining the analyses of Table 4 and Table 5 and comparing the results of Zeng’s [49] freeze–thaw experiments on two types of concrete with and without basalt fibers, it can be found that the P_0_-value of concrete doped with basalt fibers is basically the same as that of concrete without basalt fibers, but there is an increased difference in the K-value, which is explained by the fact that the concrete with basalt fibers has a lower capacity to develop defects. It indicates that the addition of basalt fibers reduces the ability of the concrete to develop defects, i.e., the admixture of basalt fibers improves the frost resistance of concrete, which is in agreement with the conclusions obtained by Jin [54]. In addition, by the comparison of two sets of experimental data from Wei [53] on concrete with and without recycled coarse aggregate, it can be observed that the K-value of concrete with recycled coarse aggregate is basically the same as that of unadulterated concrete, which means that the ability to develop defects within the two types of concrete is basically the same. However, the P_0_-value of the concrete with recycled coarse aggregate increased significantly, implying that the inclusion of recycled coarse aggregate increases the initial defects in concrete, which is consistent with the conclusions drawn by Zaharieva [55]. The findings above also further justify the physical significance of parameters K and P_0_ in the freeze–thaw damage model developed in this study.

## 6. The Comparison Between the Proposed Model and Similar Models

**Figure 13:**
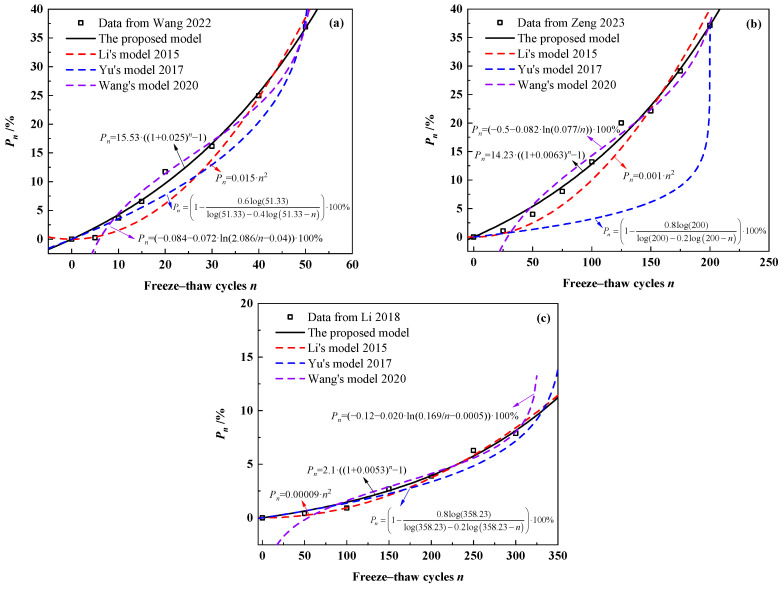
Comparison among concrete freeze–thaw damage models [20,22,25] based on (**a**) data from Wang [46]; (**b**) data from Zeng [51]; and (**c**) data from Li [18].

In order to further confirm the accuracy and applicability of the model in this study, the model was compared with other models from previous studies, outlined in Table 1, by verifying them with experimental data. Given that some parameters of the Wang [23] and Qu [26] models in Table 1 are difficult to test, the other three models were chosen to compare with the model of this study, and the models were fitted using the experimental data of Wang [46], Zeng [51] and Li [18]. Figure 13 illustrates the fitting results of the four prediction models. As shown in Figure 13, both the model proposed in this study and the other three models were able to describe the loss of relative dynamic elastic modulus of concrete during freeze–thaw cycles. It is worth noting that there is a significant deviation in fitting Zeng’s test data with Yu’s model.

**Figure 14:**
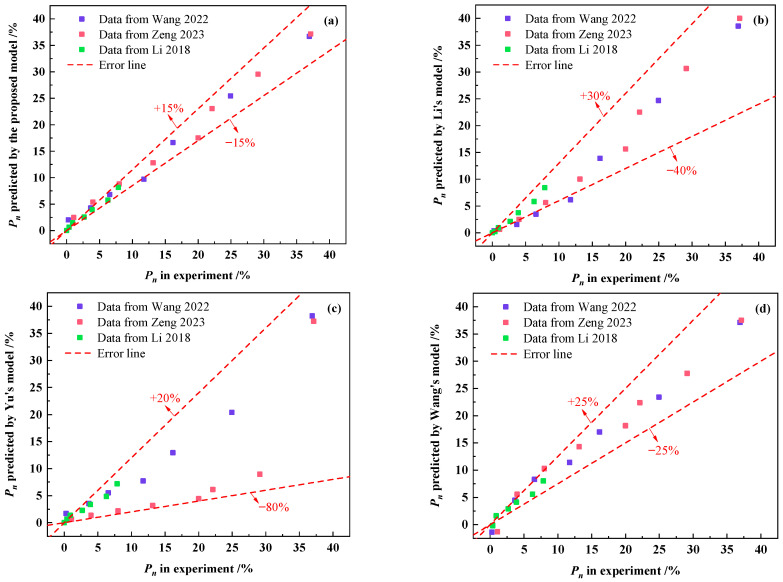
Relative errors between the Pn from research studies [18,46,51] and the P_n_ predicted by (**a**) the prediction model proposed in this work; (**b**) Li’s model; (**c**) Yu’s model; and (**d**) Wang’s model.

Figure 14 demonstrates the relative errors between the experimental data of the measured relative dynamic elastic modulus loss of Wang [46], Zeng [51] and Li [18] and the predicted values obtained by calculating with the four models. As depicted in Figure 14, the relative errors predicted by the freeze–thaw damage model proposed in this study can be controlled within ±15%, while the relative errors predicted by the other three models are above ±25%. Therefore, it can be seen that the freeze–thaw damage model for concrete proposed in this study has higher accuracy and applicability.

## 7. Conclusions

In this paper, a new concrete freeze–thaw damage model was proposed based on the hydraulic pressure theory, and the change law of the dynamic elastic modulus loss in the freeze–thaw cycle of concrete was quantified. Freeze–thaw tests were carried out on concrete containing different amounts of air-entraining agent, and the model proposed in this study was verified using the data obtained from this test and the test data of other scholars. The specific conclusions are as follows:

(1) A new freeze–thaw damage model was established based on the hydraulic pressure mechanism. The total amount of defects inside concrete is related to the initial defects inside concrete and the ability of defect development.
(2) Based on the defect evolution equation proposed in this paper, the relative dynamic elastic modulus loss equation is established. It can be seen from the equation that the loss of relative dynamic elastic modulus increases exponentially during the freeze–thaw cycle.
(3) The relative dynamic elastic modulus loss equation of concrete during freeze–thaw cycle was established considering the change of air content in concrete. The higher the air content of concrete, the smaller the parameters K and P_0_ in the dynamic elastic modulus damage equation.
(4) The model established in this study is suitable for describing the loss of relative dynamic elastic modulus of various types of concrete during the freeze–thaw process under different freeze–thaw conditions and is universally adaptable.
(5) The defect development capacity (K) in the model ranges from 0.001 to 0.050, and the initial loss of relative dynamic elastic modulus (P_0_) ranges from 0.1 to 100 (%).

The model proposed in this paper is completely based on the hydraulic pressure theory, and the influence of other freeze–thaw damage theories is not considered at present. In the future, researchers could consider more different types of defect development modes in the freeze–thaw damage model.

## Figures and Tables

**Table 1:** Summary of concrete freeze–thaw damage models.

Researchers Reference	Freeze–Thaw Damage Models	Parameters
Yan Li 2015 [20]	Dn=12⋅eg⋅n2	*D_n_*: loss of relative dynamic elastic modulus *e_g_*: relative dynamic elastic modulus acceleration *n*: number of freeze–thaw cycles
Hongfa Yu 2017 [22]	$\begin{array}{l} D_{n}=1-\frac{1-\beta \log N}{1-\beta \log\left(N-n\right)}\;(0\leq n\leq N-1)\\ \beta =\frac{D_{N-1}}{\log N} \end{array}$	*D_n_*: loss of relative dynamic elastic modulus *N*: the standard fatigue life of concrete subjected to freeze–thaw cycles *n*: number of freeze–thaw cycles *β*: concrete material parameter
Xiaoxiao Wang 2018 [23]	$\begin{array}{l} D_{n}=1-{\left[{\left(1-D_{0}\right)}^{\beta +1}-\frac{C\left(\beta +1\right)\sigma_{\max}^{\beta }}{E_{0}^{\beta }}N\right]}^{\frac{1}{\beta +1}}\\ \beta =\frac{\lambda -\frac{E_{t}}{E_{0}}}{1-D_{0}-\lambda };\;C=\frac{1-D_{0}-\lambda }{\varepsilon_{t}^{\beta }};\;\\ \lambda =\frac{\sigma_{t}}{E_{0}\cdot \varepsilon_{t}};\;\sigma_{t}=\sigma {|}_{\varepsilon =\varepsilon_{t}}\; \end{array}$	*D_n_*: loss of relative dynamic elastic modulus *D*_0_: initial damage of the concrete at the time of loading *C*: maximum strain of the concrete prior to damage localization *β*: material parameters *σ_max_*: maximum hydraulic pressure *ε_t_*: strain value where the stress is 80% of the concrete tensile strength *E*_0_: initial elastic modulus of the concrete *E_t_*: tangent modulus when the strain is *ε_t_*
Boxin Wang 2020 [25]	$D_{n}=\alpha -\beta \ln(\frac{k}{n}-\gamma )$	*D_n_*: loss of relative dynamic elastic modulus *n*: number of freeze–thaw cycles *α*; *β*; *γ*; *k*: undetermined parameters that can be confirmed by test
Guanglei Qu 2023 [26]	$D_{n}=1-\exp\left[-{\left(\frac{-\frac{\ln\frac{F(n)}{F_{0}}}{k}-\mu }{\alpha }\right)}^{\beta }\right]$	*D_n_*: loss of relative dynamic elastic modulus *n*: number of freeze–thaw cycles *F*(*n*): strength of concrete after n freezing and thawing *F*_0_: initial strength of concrete *k*: strength loss rate *α*: empirical parameter *β*: empirical parameter *μ*: empirical parameter

**Table 2:** Mix proportion of concrete.

Mixture Notation	Group	Air Content (%)	W/C	Mix Proportion (kg/m^3^)				Sand Ratio
				Water	Cement	Sand	Coarse Aggregate	
Ordinary Portland concrete	A	0.8	0.45	195.0	435.0	566.7	1204.3	0.32
Air-entrained concrete	B	2.7						
	C	4.5						
	D	6.6						

**Table 3:** Parameter of the relative dynamic elastic modulus loss equation for different groups.

Group	Mixture Notation	Pn = P_0_ ((1 + k)^n^ − 1)			Air Content (%)
		P_0_	K	R^2^	
A	Ordinary Portland concrete	9.05	0.0161	0.99	0.8
B	Air-entrained concrete	2.96	0.0124	0.99	2.7
C		2.40	0.0100	0.99	4.5
D		1.35	0.0056	0.99	6.6

**Table 4:** Test data of the loss of relative dynamic elastic modulus from researchers.

n ^1^	The Loss of Relative Dynamic Elastic Modulus (%)										
	Wang [46]	Xiao [48]	Zeng [49]		Xiao [21]	Alrikabi [50]	Zeng [51]	Jiang [52]	Wei [53]		Li [18]
0	0.00	0.00	0.00	0.00	0.00	0.00	0.00	0.00	0.00	0.00	0.00
5	0.26	-	-	-	-	-	-	-	-	-	-
10	3.68	-	-	-	-	-	-	-	-	-	-
15	6.57	-	-	-	-	-	-	-	-	-	-
20	11.74	-	-	-	-	-	-	-	-	-	-
25	-	-	-	-	0.83	-	1.09	3.24	0.20	1.60	-
30	16.17	-	-	-	-	12.48	-	-	-	-	-
35	-	-	-	-	-	-	-	-	-	-	-
40	24.95	-	-	-	-	-	-	-	-	-	-
50	36.92	2.99	2.92	1.89	1.37	-	4.00	14.69	0.95	3.10	0.40
60	-	-	-	-	-	28.19	-	-	-	-	-
75	-	-	-	-	2.10	-	8.01	21.93	2.58	5.12	-
80	-	-	-	-	-	-	-	-	-	-	-
90	-	-	-	-	-	44.9	-	-	-	-	-
100	-	8.98	6.24	4.26	3.00	-	13.17	41.70	3.85	6.53	0.91
120	-	-	-	-	-	70.08	-	-	-	-	-
125	-	-	-	-	3.18	-	20.02	-	4.96	8.61	-
150	-	18.00	13.45	7.42	4.48	-	22.13	-	6.13	10.47	2.69
175	-	-	-	-	6.00	-	29.12	-	7.50	12.72	-
200	-	30.96	23.60	11.53	6.68	-	37.13	-	8.39	15.86	3.89
225	-	-	-	-	7.52	-	-	-	9.43	19.12	-
250	-	41.98	42.24	19.30	10.77	-	-	-	11.16	21.79	6.28
275	-	-	-	-	15.79	-	-	-	13.64	25.94	-
300	-	-	-	29.13	23.81	-	-	-	15.53	29.04	7.88
Freeze–thaw condition	Pure water	Pure water	Pure water	Pure water	Pure water	Pure water	3.5% NaCl	6% NaCl 3% Na_2_SO_4_	5% Na_2_SO_4_	5% Na_2_SO_4_	5% Mg_2_SO_4_
W/C	0.5	0.45	0.29	0.29	0.45	0.32	0.50	0.45	0.38	0.38	0.5
Adulteration	/	RCA ^2^	Lytag ^3^	Lytag BF ^4^	RCA	FA ^5^	FA SF ^6^	FA	FA	FA RCA	/
Admixtures	/	WRA ^7^ AEA ^8^	WRA	AEA	WRA	WRA	WRA	WRA AEA	WRA AEA		

^1^ The number of freeze–thaw cycles. ^2^ Recycled coarse aggregate. ^3^ Fly ash lightweight aggregate. ^4^ Basalt fiber. ^5^ Fly ash. ^6^ Steer fiber. ^7^ Water-reducing agent. ^8^ Air-entraining agent.

**Table 5:** Value of parameters in relative dynamic elastic modulus loss Equation (9).

	Wang [46]	Xiao [48]	Zeng [49]		Xiao [21]	Alrikabi [50]	Zeng [51]	Jiang [52]	Wei [53]		Li [18]
P_0_(%)	15.53	12.78	3.73	3.64	0.74	49.54	14.23	8.67	9.18	14.97	2.10
K	0.0246	0.0059	0.0101	0.0074	0.0115	0.0073	0.0064	0.0177	0.0033	0.0036	0.0053
Correlation (R^2^)	0.99	0.99	0.99	0.99	0.97	0.99	0.99	0.99	0.99	0.99	0.99

## Data Availability

The original contributions presented in this study are included in the article. Further inquiries can be directed to the corresponding author.

## Abbreviations

The following abbreviations are used in this manuscript:

OPC	Ordinary Portland cement
DEF	Defects
ND	New defects
RCA	Recycled coarse aggregate
BF	Basalt fiber
FA	Fly ash
SF	Steer fiber
WRA	Water-reducing agent
AEA	Air-entraining agent
